# Advanced Gene-Targeting Therapies for Motor Neuron Diseases and Muscular Dystrophies

**DOI:** 10.3390/ijms23094824

**Published:** 2022-04-27

**Authors:** Myrsini Chamakioti, Nikolaos Karantzelis, Stavros Taraviras

**Affiliations:** 1 Department of Physiology, School of Medicine, University of Patras, 26504 Patras, Greece; m.chamakioti@gmail.com (M.C.); nikolaos.karantzelis@uniklinik-freiburg.de (N.K.); 2 Department of Hematology, Oncology and Stem Cell Transplantation, University Medical Center Freiburg, 79106 Freiburg, Germany

**Keywords:** gene-targeting therapy, motor neuron disorders, muscular dystrophies

## Abstract

Gene therapy is a revolutionary, cutting-edge approach to permanently ameliorate or amend many neuromuscular diseases by targeting their genetic origins. Motor neuron diseases and muscular dystrophies, whose genetic causes are well known, are the frontiers of this research revolution. Several genetic treatments, with diverse mechanisms of action and delivery methods, have been approved during the past decade and have demonstrated remarkable results. However, despite the high number of genetic treatments studied preclinically, those that have been advanced to clinical trials are significantly fewer. The most clinically advanced treatments include adeno-associated virus gene replacement therapy, antisense oligonucleotides, and RNA interference. This review provides a comprehensive overview of the advanced gene therapies for motor neuron diseases (i.e., amyotrophic lateral sclerosis and spinal muscular atrophy) and muscular dystrophies (i.e., Duchenne muscular dystrophy, limb-girdle muscular dystrophy, and myotonic dystrophy) tested in clinical trials. Emphasis has been placed on those methods that are a few steps away from their authoritative approval.

## 1. Introduction

Neuromuscular diseases are characterized by impaired function of the motor unit components, causing significant motor disorders and leading to respiratory insufficiency and early death in the most adverse clinical cases. Many of these clinical entities are caused by inborn genetic abnormalities and progress irreversibly.

Until recently, the existing clinical approach could not address the root cause of the disease, leading in many cases to unfavorable clinical outcomes and thus highlighting the inevitable need for alternative forms of therapies, which could eventually contribute to a more target-specific approach.

The genomic revolution accomplished over the last decades facilitated the identification of several disease-causing genes and thereby contributed to the development of targeted clinical approaches in the form of gene therapies.

By using the term *gene therapies*, we refer to therapies that act at the level of deoxyribonucleic acid (DNA) or ribonucleic acid (RNA) and can theoretically “correct” any type of mutation. The major types of genetic treatments that apply to today’s clinical practice are summarized below.

### 1.1. Gene Replacement Therapy (GRT)

The basic idea behind GRT is simple: the transfer of corrective genetic material into cells alleviates disease symptoms [1]. The therapeutic genomic cargo includes the therapeutic gene, a promoter, and regulatory elements, and it is delivered to the affected cell through a vector. Intracellularly, the transgene uses the transcriptional and translational machinery of the own cell and repetitively synthesizes its products, addressing a loss-of-function mutation and providing a long-term therapeutic effect [2].

The enhancement of the transgene expression and the therapeutic safety of GRT are highly dependent on the selection of the ideal vector. This selection is based on the type of targeted tissues and the size of the therapeutic transgene [2,3]. The vectors are divided into two major categories: viral and non-viral.

Viral delivery systems use replication-deficient, recombinant viral vectors, where the viral DNA is replaced by an expression cassette containing the therapeutic gene. The most frequently used viruses are retroviruses, adenoviruses, and adeno-associated viruses (AAVs). Retroviruses or lentiviral vectors are recombinant vectors derived from the human immunodeficiency virus. With a packaging capacity of 8–10 kb, they deliver large DNA cargos and integrate them into the host cell genome. While this results in prolonged transgene expression, it also carries the risks of genotoxicity and insertional mutagenesis [2]. AAVs are small, non-enveloped viruses with a low immunogenicity profile [3]. They have a 4.7 kb long, linear, single-strand DNA genome, restricting GRT application to small transgenes. Some AAV serotypes, such as AAV2, show neuronal tropism and sustained neuronal expression, whereas AAV9 and AAVrh.10 can penetrate the BBB [4,5,6,7]. AAV-based gene therapies can be administered systematically or in a targeted way, directly into the affected tissue. As expected, the systematic delivery of AAV vectors requires higher dosages (maximum dosage of 1.5 × 10^17^ viral genome [vg]) than the targeted administration (maximum dosage of 7.5 × 10^15^ vg) [8].

Non-viral gene carriers comprise the widely applied lipid-based vectors and the emerging cationic polymeric vectors. In contrast to viral vectors, non-viral vectors neither have a gene size limitation nor trigger an immune response. However, their transient gene expression requires that they are administrated in high doses. For this reason, viral vectors are mostly preferred [2,9].

### 1.2. Antisense Oligonucleotides (ASOs)

An ASO is a synthetic nucleic acid strand designed to hybridize to an RNA target via Watson–Crick base pairing and modulate gene expression. An ASO binds to the RNA and acts with one of two major mechanisms presented below. The first mechanism involves promoting the endo-nucleolytic cleavage of the target. RNase H1 binds to the heteroduplex of the ASO with the messenger RNA (mRNA) and cleaves the mRNA, reducing the corresponding protein. RNase-mediated degradation is particularly advantageous in disorders in which a distinct RNA/protein dysregulation or accumulation is recognized as the central cause of disease [10]. ASOs currently undergoing trials in amyotrophic lateral sclerosis (ALS) belong to this category. Based on the second mechanism, an ASO acts by sterically blocking the binding of trans-acting factors (e.g., RNA-binding proteins and non-coding RNAs). The ASOs work as splice switching oligonucleotides and manipulate alternative pre-mRNA spicing. ASOs block RNA sequences essential for splicing and prevent the interaction of splicing factors—such as RNA-binding proteins, small nuclear RNAs, and other spliceosome components—with the pre-mRNA [10]. Within this framework, ASOs can be employed to induce exon inclusion (spinal muscular atrophy [SMA]) or exon skipping (familial ALS [fALS]) and restore the mRNA reading frame (Duchenne muscular dystrophy [DMD]).

Unmodified ASOs suffered from poor solubility and rapid degradation by exonucleases, which led to the development of the so-called first-generation ASOs with substitutions of the phosphate backbone linking the nucleotides. The most remarkable and widely used chemical alteration is also the oldest: the phosphorothioate (PS) modification [11]. Enhanced interactions of PS ASOs with serum proteins protect ASOs from nucleases and reduce their renal clearance, thereby stabilizing the serum concentration of ASOs. However, changing the achiral phosphodiester center into a chiral PS center at every linkage along the ASO doubles the number of possible stereoisomers. Whether controlling this PS chirality (stereopure ASOs) or not (stereorandom ASOs) affects the therapeutic profile of ASOs is still being investigated. Iwamoto and colleagues provided evidence that some stereopure PS ASOs are better recognized by RNase H [12], but Østergaard and colleagues concluded that there is not enough evidence that PS ASOs improve the overall therapeutic profile in a meaningful manner [13]. Nevertheless, stereopure ASOs have been advanced to clinical trials, and, at the time of writing, a stereopure ASO is being tested in a phase 1 trial for ALS and frontotemporal dementia (FTD).

In addition to the PS backbone modification, recent pharmacology includes oligonucleotides that are further modified at the 2′ position of the sugar ring, also known as second-generation ASOs. Currently, the most widely used 2′ modifications in ASO therapeutics are the 2′-O-Methyl (2′-O-Me) and the 2′-O-Methoxyethyl (2′-O-MOE) substitutions [14]. Especially for the MOE side chain, it improves ASOs’ nuclease resistance and hybridization affinity to the complementary RNA. However, 2′-O-MOE ASOs have a limited ability to activate RNase H, which cleaves the target mRNA. Thus, this is achieved using the “gapmer” approach, where a central core of PS ASOs is flanked by outer wings of 2′-O-MOE oligonucleotides. The resulting gapmer combines the ability of the PS modification to activate RNase H with the increased hybridization affinity and nuclease resistance of the 2′-O-MOE modification [15].

In more recent iterations, the so-called third-generation ASOs aim to improve even further the pharmacokinetic profile and potency of ASOs and include a wide variety of molecules, such as locked nucleic acids, N-methyl substituted bicyclic nucleic acids, and peptide nucleic acids. Among them, the most widely used are the phosphorodiamidate morpholino oligomers—also called morpholinos—(PMOs), which have a morpholine ring instead of a ribose ring, and neutral phosphorodiamidate linkages instead of charged phosphodiester linkages. PMOs are mainly used as splice-switching ASOs, as their non-natural structure does not support RNase H function. Their major advantage is that they are very safe; they can be administered in high doses as their uncharged backbone results in low protein binding.

### 1.3. RNA Interference (RNAi)

RNAi is a phenomenon of post-transcriptional gene silencing induced by small, non-coding double-stranded RNAs (dsRNAs) [16,17]. In general, non-coding RNAs are molecules that modulate processes such as chromatin remodeling, transcription, and signal transduction. They function as crucial regulators of both developmental pathways and pathogenic procedures, such as cancer [18]. Three types of small non-coding RNAs trigger RNAi: microRNA (miRNAs), small interfering RNAs (siRNAs), and piwi-interacting RNAs (piRNAs). miRNAs and siRNAs play a role as negative regulators of gene expression, while the piRNAs defend organisms against transposable elements.

The basic idea behind RNAi is that, physiologically, the introduction of long dsRNA into cells leads to the sequence-specific degradation of homologous gene transcripts. The dsRNA, either transcribed from infecting pathogens [19] or artificially introduced into the cells, is processed by Dicer, a specialized RNase III-like enzyme, into a smaller dsRNA molecule. This short dsRNA molecule is known as the siRNA and has 21–23 nucleotides with 3′ two-nucleotide overhangs. The siRNA interacts with and loads into the RNA-induced silencing complex (RISC). The endonuclease argonaute 2 (AGO2) component of the RISC cleaves the siRNA’s passenger strand (sense strand). The guide strand (antisense strand) remains associated with the RISC. Subsequently, the guide strand guides the active RISC to mRNAs bearing complementary target sites. The complementary binding activates the AGO2, which then cleaves the mRNA 10 and 11 nucleotides downstream from the 5′ end of the antisense strand [20]. The generated mRNA fragments are subsequently degraded by different exonucleases. As the guide strand only binds to entirely complementary mRNA, siRNA causes specific gene silencing.

### 1.4. CRISPR-Associated (Cas) Systems

A CRISPR-associated (Cas) system, also known as genetic scissors, is a gene-editing system in which an RNA-guided Cas9 endonuclease (CRISPR-Cas9) inducts double-stranded DNA breaks in the genomic sequences, causing frameshift-inducing base insertions or deletions [21,22]. Early-stage clinical trials of CRISPR-Cas9 are underway in blood disorders [23], cancers [24], eye diseases [25], and protein-folding disorders [26]. Unlike the previous three methods, there has been no CRISPR clinical trial for motor neuron diseases and muscular dystrophies until today. Nonetheless, its success in pre-clinical studies makes it an up-and-coming candidate soon entering the clinical horizon [27].

The above-presented genetic treatments can theoretically “correct” any type of mutation, overcoming unfavorable clinical outcomes associated with resistance to conventional drug therapy. In addition, they have long-lasting therapeutic effects. Thanks to these favorable properties, they find application in a wide range of fields, including inherited genetic disorders such as hemophilia [28] and cystic fibrosis [29], or acquired diseases, such as cancer [30], infectious diseases, and acquired immunodeficiency syndrome (AIDS) [31].

In addition, gene therapy is of great interest for central nervous system (CNS) diseases. It is particularly attractive for muscular dystrophies and motor neuron diseases because, in some disease types, there is a lack of disease-targeting drugs, while in others, the few established targeted treatments have failed to demonstrate clinical efficacy. For example, despite the significant therapeutic advances over the past 30 years in muscular dystrophies, the treatment was based on multidisciplinary management of symptoms that improved quality of life [32]. This was also the case for motor neuron diseases; for SMA, treatment was symptomatic, while for ALS, only edaravone [33,34,35] and riluzole had slowed progression, both of them moderately and by targeting non-specific factors [36,37].

Recent advances in our understanding of the pathophysiology of these diseases, including the discovery of their genetic underpinnings, enabled the development of targeted, efficacious treatments. The most representative example is SMA: administration of nusinersen—an ASO approved in 2016 [38]—during the presymptomatic period has transformed SMA from a prematurely fatal disease to one with almost physiological development [39]. Given that new successful therapeutic strategies in one disease often inspire research paths in related ones, the development of the first gene therapies led to the investigation of similar approaches in ALS and muscular dystrophies. Remarkably, since 2017, four ASOs have been approved for DMD, illustrating the unprecedented therapeutic potential of genetic treatments [40,41,42,43].

**Figure 1:**
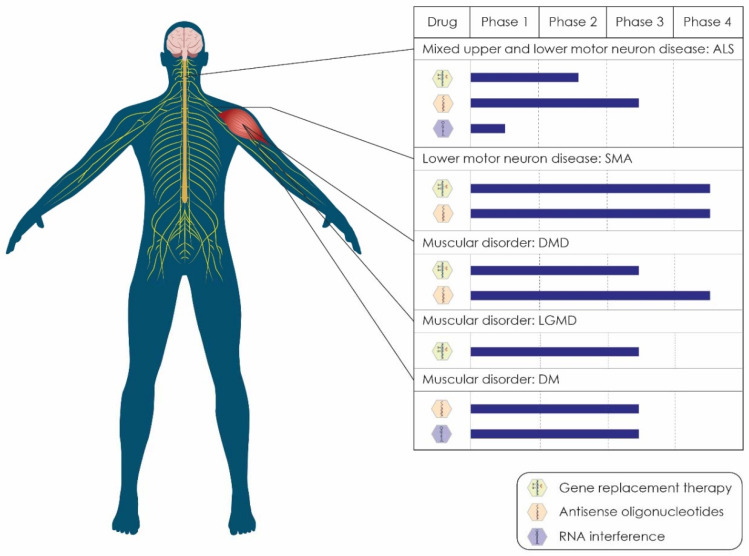
Status of the most advanced gene therapies for motor neuron diseases and muscular dystrophies. In ALS, in which the pathology is located both in upper and lower motor neurons, the most advanced GRT has reached phase 2, the most advanced ASO has reached phase 3, and the most advanced RNAi drug has reached phase 1. In SMA, a disease of the lower motor neurons, both a GRT and an ASO, have gained FDA approval and are currently tested in phase 4 clinical trials. In DMD, a disorder that targets the muscles, GRTs are presently tested in phase 3 clinical trials, whereas ASOs have gained FDA approval. In LGMD, which is also a muscle disorder, GRTs are currently tested in phase 3 clinical trials. In DM, both ASOs and RNAi drugs have reached phase 3 of clinical trials. Abbreviations: ALS, amyotrophic lateral sclerosis; GRT, gene-replacement therapy; ASO, antisense oligonucleotide; RNAi, RNA interference; SMA, spinal muscular atrophy; DMD, Duchenne muscular dystrophy; LGMD, limb-girdle muscular dystrophy; DM, myotonic dystrophy.

The purpose of this review is to provide an in-depth overview of the current status of the landscape of gene therapy clinical trials for motor neuron diseases and muscular dystrophies. Emphasis has been placed on the genetic background of clinical entities, including ALS, SMA, DMD, limb-girdle muscular dystrophy (LGMD), and myotonic dystrophy (or dystrophia myotonic) (DM), and the corresponding gene-targeting therapeutic approaches. We have grouped the diseases mentioned above based on their pathophysiology and the status of gene therapy treatments currently evaluated in clinical trials (Figure 1). We have also discussed the existing limitations regarding the application of gene therapy as a standard way of treatment.

## 2. Amyotrophic Lateral Sclerosis (ALS)

### 2.1. Clinical Manifestations and Genetic Background

ALS is a rare and incurable neurodegenerative disease, also known as Lou Gehrig’s disease, after the famous baseball player was diagnosed with it in 1939 [44]. The main feature of the disease is the selective death of motor neurons in the spinal cord, the brainstem, and the primary motor cortex [45]. The loss of upper motor neurons leads to symptoms such as spasticity and hyperreflexia, whereas the loss of lower motor neurons leads to fasciculations, muscle weakness, and atrophy [44]. As the disease progresses, bulbar symptoms may occur, such as dysphagia and dysarthria, leading to malnourishment and sialorrhea [45]. In addition to motor symptoms, cognitive impairment can happen; there can be behavioral disturbances, irritability, and FTD [46]. The clinical phenotype and the progression of the disease may vary considerably between patients. However, all patients will eventually pass away, typically 2 to 5 years after the onset of the disease and usually from respiratory insufficiency [47].

Among patients with ALS, 90–95% have the sporadic type of the disease, while the familial type (fALS) is observed only in 5–10% of cases. Although, in most cases, the cause is not known, environmental and genetic factors have been linked to the disease [44]. Regarding the genetic factors, more than 30 genes are associated with the disease [44]. Mutations in superoxide dismutase 1 (SOD1), fused in sarcoma (FUS), chromosome 9 open reading frame 72 (C9orf72), and transactive response DNA-binding protein 43 (TDP-43) have the highest prevalence [44]. Several gene therapies for many of these ALS types are currently tested in clinical trials (summarized in Table 1).

### 2.2. Available Gene Therapies for ALS

#### 2.2.1. GRT in ALS

Unlike other diseases, ALS does not have an exclusive loss-of-function underlying mechanism. In addition, the cerebral cortex and the anterior horn cells of the spinal cord, which are affected by ALS, are some of the most challenging parts of the body to reach. The approved drugs may show some initial blood-brain barrier (BBB) permeability, but their therapeutic efficacy is limited due to pharmacoresistance that develops over the course of ALS and their inability to target the genetic cause of the disease. Thus, methods of gene therapy, such as GRT, ASOs, and RNAi, are very promising therapeutic approaches, as (a) they can reach the brain and spinal cord via minimally invasive means, (b) they directly target the causative genes of the disease, and (c) they offer a long-lasting effect [48].

Due to the complex genetics of ALS, GRT cannot cure the disease by providing only one functional gene. However, it can prolong the lifespan and ameliorate the symptoms of the disease by providing the degenerated neurons with genes that encode neurotrophic factors. Neurotrophic factors promote the survival of all types of neurons, thus acting beneficially for every type of ALS patient [48]. A wide range of neurological factors has been shown to attenuate motor neuron death in ALS animal models. One of them, the hepatocyte growth factor (HGF), which has neurotrophic and angiogenetic properties, was advanced in clinical trials. On March 10, 2021, Helixmith announced the enrollment of the first patient in a phase 2, double-blind, randomized clinical trial (namely, REViVALS-1A), which aims to evaluate the safety and tolerability of an HGF-containing GRT named Engensis in 18 ALS patients (NCT04632225).

Helixmith’s Engensis consists of the HGF coding sequence integrated into a plasmid DNA. It expresses two isoforms of HGF (HGF_728_ and HGF_723_) [49], which, when combined, boost the angiogenetic and neurotrophic activities of HGF (formation of collateral blood vessels in animal diabetic models [50] and promotion of neuronal survival and axonal outgrowth [51,52,53,54,55,56,57,58,59,60]). The safety of Engensis has already been proven by clinical studies on different diseases, such as painful diabetic peripheral neuropathy [61] and critical limb ischemia [62]. Currently, apart from the phase 2a REViVALS-1A trial, there is a 6-month extension study, which aims to evaluate the long-term safety of Engensis (NCT05176093). If the currently ongoing trials support the efficacy and safety of Engensis, Helixmith will conduct an expanded phase 2b trial [63].

#### 2.2.2. ASOs in ALS

Another promising tool to target the toxic gain of function mechanisms responsible for certain forms of fALS is ASOs. Below, we provide an overview of ongoing clinical trials using ASOs for ALS (see Table 1).

##### An ASO RNase H1-Mediated Inhibitor of SOD1: Tofersen

The first gene that acted as a target for ASOs in neurodegenerative diseases was also the first one to be associated, in 1993, with ALS: SOD1 [64]. Responsible for 20% of fALS cases, mutations in the SOD1 gene cause the abnormal aggregation, dimer destabilization, and oligomerization of the SOD1 protein. Subsequently, they lead to oxidative stress and neurotoxicity [65]. The knowledge of this gain-of-function mechanism of toxicity made the SOD1 gene an ideal target for antisense therapeutic approaches.

Thus, in January 2010, and after its safety and efficacy were proven by extensive laboratory studies, the first ASO targeting SOD1 entered clinical trials. ISIS333611 was an ASO that degraded SOD1 transcripts through RNase H and was administered via a 12-h intrathecal (IT) infusion during a first-in-human, phase 1 clinical study by Ionis Pharmaceuticals (NCT01041222). The low doses of ISIS333611 were not considered efficacious; therefore, the Ionis’ phase 1 clinical trial was terminated. Although the primary efficacy endpoint was not achieved, this was the first study that proved the safety of IT administration of an ASO into the CNS, preferably via bolus injection (which proved to be more efficient than the constant, 12 h injection) [66]. Therefore, it was decided that an extensive screening in cell culture and transgenic rodents was required before administering a drug via bolus IT infusion. Such a drug is tofersen, an ASO RNase H1-mediated inhibitor of SOD1 mRNA. In January 2016, a phase 1/2 ascending-dose trial of tofersen began by Ionis and Biogen. The results of this study were promising and revealed that patients that had been administered the highest concentration of tofersen had a 36% decline in SOD1 protein levels in the cerebrospinal fluid (CSF). Regarding safety, except for the CSF pleocytosis in some patients for an unknown reason, most adverse events were ascribed to the lumbar puncture procedure [67].

The overall encouraging results allowed the promotion of tofersen to a phase 3, randomized, double-blind, placebo-controlled trial named VALOR (NCT02623699), which aimed to evaluate the safety and efficacy of tofersen, along with its long-term extension study (NCT03070119). Tofersen did not show a statistically significant change from baseline to week 28 in the Revised Amyotrophic Lateral Sclerosis Functional Rating Scale. As a result, it did not meet the primary endpoint. However, the ASO met multiple exploratory and two critical secondary endpoints; in comparison to placebo groups, it showed a reduction in the total CSF SOD1 protein (38% and 26% in the faster- and slower-progressing populations, respectively) and a reduction in the plasma levels of neurofilament light chain, a potential marker of neuronal degeneration (67% and 48% in the faster- and slower-progressing populations, respectively). In addition, as shown by a pre-specified integration of data from VALOR and its ongoing open-label extension (OLE) study, initiation of tofersen in the early disease stages reduces the deterioration of motor function, respiratory function, muscle strength, and quality of life in people with SOD1-ALS [68].

After evaluating the above results, Biogen decided to expand its ongoing early access program (EAP) to all SOD1-ALS patients in countries where such programs are permitted. In the future, when more data regarding tofersen are available, Biogen may reevaluate this EAP.

Apart from treating ALS patients with symptomatic disease, another exciting aspect is the early intervention in ALS before symptoms appear. Therefore, tofersen is currently being tested in pre-symptomatic adult carriers of a SOD1 mutation with elevated neurofilaments in a phase 3 trial named ATLAS (NCT04856982). The study, initiated in May 2021, is conducted in France and the United States and involves 150 participants.

##### An ASO RNase H1-Mediated Inhibitor of ATXN2 mRNA: BIIB105

Long polyglutamine (polyQ) repeats (CAG trinucleotide repeat expansions) in the ataxin-2 (ATXN2) gene may cause spinocerebellar ataxia [69], but intermediate polyQ repeats (27 to 33 CAG trinucleotide repeat expansions) have been associated with sporadic ALS [70].

In 2006, it was found that mutations in the TAR DNA-binding protein (TARDBP) gene, which encodes the TDP-43 protein, are responsible for familial cases of ALS and frontotemporal lobar degeneration with TDP-43 inclusions (FTLD-TDP) [71,72]. TDP-43 protein is an RNA-binding protein that is physiologically localized in the nucleus. In ALS and FTLD-TDP, mutated TDP-43 toxically aggregates in ubiquitinated cytoplasmic inclusions (stress granules), pathologically concentrated in patients’ spinal cord neurons [73]. By binding key factors for nucleocytoplasmic transport, such as karyopherins, stress granules disrupt the nucleocytoplasmic shuttling and cause cell death [74,75].

Importantly, preclinical studies have shown that this TDP-43 toxicity is exacerbated by the ATXN2 protein. ATXN2 and TDP-43 are both involved in RNA metabolism by forming an RNA-dependent complex in the cytoplasm [76]. In ALS, the polyQ repeats of ATXN2 enhance the protein-protein interaction between ATXN2 and TDP-43 and promote the formation of stress granules by TDP-43 [75].

It should be pointed out that TDP-43 pathology is a crucial disease mechanism not only in familial but also in sporadic ALS cases. Consequently, an ASO that would mediate TDP-43 toxicity by targeting ATXN2 would benefit a vast population of ALS patients.

Such an example would be BIIB105 by Ionis Pharmaceuticals. Also known as ION541, this ASO targets ATXN mRNA and induces its degradation through RNase H1. Based on its promising results in preclinical studies regarding lifespan, motor function, and the onset of symptoms, a phase 1, triple-blind, multiple-ascending-dose clinical trial was initiated in September 2020 (NCT04494256). The study evaluates the safety, tolerability, and pharmacokinetics of BIIB105 in patients both with and without CAG repeat expansions in ATXN2. It is being conducted at nine sites in the United States, Canada, and the Netherlands, and it is estimated to be completed in February 2023.

##### An ASO RNase H1-Mediated Inhibitor of C9ORF72 RNA: BIIB078

The gene that is most frequently mutated in ALS (34% of all fALS and 12% of all ALS cases), as well as in FTD, is C9orf72, on the short arm of chromosome 9. This gene normally produces a protein present in a large percentage of neurons and regulates many cellular functions, such as endocytosis and autophagy [77]. In ALS, expansion of the GGGGCC hexanucleotide sequence is detected in two of the gene’s three transcript variants (V1 and V3), resulting in the loss of a significant amount of normal protein [78,79]. In the nucleus, the mutated variants aggregate as toxic RNA foci. In the cytoplasm, the mutated sense and antisense variants are translated into toxic dipeptides, called dipeptide repeat proteins, which form clumps. Eventually, the toxic effects of the C9orf72 mutant gene products lead to neuronal death [80,81].

Aiming to inhibit the toxic effect of mutant transcripts, Biogen, in collaboration with Ionis, developed an ASO called BIIB078, which targets GGGGCC-containing RNAs and mitigates their degradation through RNase. The production of C9orf72 protein by the normal variant is not affected. In preclinical studies, BIIB078 was shown to produce sustained reductions in RNA foci and dipeptide-repeat proteins and attenuate cognitive and behavioral deficits [82]. About 5 years later, in September 2018, the first-in-human clinical trial of BIIB078 was initiated (NCT03626012). The study primarily tested the safety and tolerability of the ASO in C9ORF72-ALS patients in a randomized, quadrable masking way, and results have not been announced yet. The extension study of the above trial is currently conducted (NCT04288856).

##### A Stereopure ASO RNase H1-Mediated Inhibitor of C9ORF72 RNA: WVE-004

Recently, Wave Life Sciences announced the discovery of another ASO to treat C9orf72-associated ALS and FTD, which was named WVE-004. The oligonucleotide sequence complementary to WVE-004 is present in every C9orf72 transcript. However, WVE-004 is a stereopure ASO that targets only HRE-containing variants (V1 and V3) and induces their degradation through RNase [83].

The preclinical study results of WVE-004 were promising. WVE-004 yielded a 6-month-sustained reduction in mutated transcripts and dipeptide repeat proteins. At the same time, it spared the V2 transcript and allowed the physiological production of normal C9orf72 [84].

Based on these results, Wave Life Sciences advanced WVE-004 to clinical trials. Since June 2021, the safety and tolerability of IT, single-ascending, and multiple-ascending doses of WVE-004 in adult patients with C9orf72-associated ALS or FTD are being evaluated in a randomized phase 1b/2a double-blind, placebo-controlled trial called FOCUS-C9 (NCT04931862). The ASO is being tested in four dose levels, and dosing is adaptable. Fluid biomarkers, functional assessments, and digital biomarkers will be reviewed by the Association for Frontotemporal Degeneration and the Alzheimer’s Drug Discovery Foundation, which support the FOCUS-C9 clinical trial.

##### A Patient-Specific ASO Targeting the FUS Mutation p.P525 L: Jacifusen

Another gene associated with ALS, particularly with early-onset and juvenile ALS, is FUS [85,86]. This gene encodes an RNA-binding protein structurally and functionally similar to TDP-43. FUS is involved in various stages of gene expression [87], DNA repair procedures [88], and cellular stress-management procedures [89].

More than 50 FUS variants have been detected related to ALS [90]. These variants encode a pathological FUS protein that accumulates inside neurons and exerts neurotoxic effects, both gain-of-function and loss-of-function. Gain-of-function mechanisms include altered RNA processing due to overexpressing of mutant FUS [91]. In contrast, loss-of-action mechanisms include altering intron retention levels in RNA-binding proteins.

To date, only one ASO targeting FUS-ALS has been advanced to clinical trials. The ASO is named ION363—also known as Jacifusen—and it was developed by Ionis Pharmaceutical in collaboration with Columbia Medical Center as a personalized gene therapy that targeted the p.P525L mutation. p.P525L mutation in the FUS gene is associated with a severe and aggressive form of FUS-ALS [92]. In preclinical studies on heterozygous mutant FUS mice, ION363 effectively silenced wild-type and mutant FUS in the brain and spinal cord [93]. Jacifusen was first tested in humans in May 2019, when it was approved by the U.S. Food and Drug Administration (FDA) for compassionate use in a 26-year-old woman with FUS-ALS whose identical twin brother had died of ALS. The woman was named Jaci, and that is where Jacifusen (*Jaci-fus-en*) has got its name [94].

Today, a phase 3 study aims to evaluate the efficacy of ION363 on clinical function and survival in carriers of FUS-ALS (NCT04768972). The study is randomized and double-blind, includes 64 participants, and will be completed by March 2024.

#### 2.2.3. RNAi in ALS

##### A miRNA Inhibitor of SOD1 mRNA: SOD1-rAAVRh10.mi-SOD1

An alternative method for ALS treatment is to silence the toxic, gain-of-function genes via RNAi. Although it is not as advanced as ASOs, RNAi has been shown to be effective in several preclinical studies [95,96,97].

The first—and only—RNAi clinical trial in ALS started in 2017 at the University of Massachusetts Medical School and Massachusetts General Hospital. The study primarily aimed to prove the safety of the RNAi treatment during its application in two patients with SOD1-mediated ALS. Patients were administered a single IT infusion of a miRNA incorporated into an AAV vector (rAAVRh10.mi-SOD1), whose nucleic acid sequence was fully complementary to the human mutated SOD1 gene. In the first patient, only a slight decrease of SOD1 levels in CSF and a transitory gain of strength in his right leg were observed. However, meningoradiculitis developed as a side effect of the miRNA IT infusion. Such an adverse inflammatory response was prevented in the second patient by administering immunosuppressant drugs. Notably, the absence of side effects was not accompanied by a significant clinical benefit [98].

## 3. Spinal Muscular Atrophy (SMA)

### 3.1. Clinical Manifestations and Genetic Background

SMA is the leading cause of infant mortality. It is an autosomal recessive disease caused mainly by mutations in the survival of motor neuron gene 1 (SMN1), one of the two genes that encode for the survival motor neuron (SMN) protein. The other gene that codes for SMN protein, SMN2, is a nearly identical copy of the SMN1 gene and remains intact in most SMA patients. However, it cannot compensate for the loss of function of the SMN protein due to a single nucleotide transition that results in the production of an unstable, poorly functional SMN protein.

From a clinical perspective, SMA is characterized by spinal motor neuron death, which gradually leads to muscle weakness and paralysis [99,100,101]. Until 2016, there was no available treatment that could change the clinical progress of this devastating disease. However, as explained below in detail, gene therapy dramatically changed SMA’s therapeutic horizon. Currently, there are several ongoing clinical trials (summarized in Table 2) evaluating the available therapies.

### 3.2. Available Gene Therapies for SMA

#### 3.2.1. GRT in SMA

The first clinical trial started in 2014 and evaluated the safety and efficacy of zolgensma, also known as onasemnogene abeparvovec–xioi, an AAV-based GRT of the human SMN gene. Zolgensma was tested in 15 SMA1 infants with two SMN copies and proved to be well-tolerated and efficient (NCT02122952). More specifically, it improved survival in treated patients compared with natural history cohorts. In addition, patients were free from permanent ventilation and achieved unprecedented motor milestones [102]. Based on these results, in May 2019, the FDA approved zolgensma [103].

Trials extended to over 100 patients, with the STR1VE trials in Europe (NCT03461289), United States (NCT03306277), and Asia Pacific (NCT03837184), the STRONG trial (NCT03381729), which tested the IT administration of the GRT, and the SPR1NT trial, which evaluated the presymptomatic treatment of SMA patients (NCT03505099). In the STR1VE trial, zolgensma increased survival and enhanced motor function in SMA infants, demonstrating a favorable benefit-risk profile [102]. Regarding the STRONG trial, although dosing was temporarily suspended due to concerns about dorsal root ganglia toxicity, zolgensma finally met the primary efficacy endpoint; in the Hammersmith Functional Motor Scale Expanded assessment, 92% of patients had a 3-point-or-greater increase in their score at a post-baseline visit [104]. In the SPR1NT trial, where zolgensma was tested in genetically diagnosed, asymptomatic SMA infants, the results were very positive. Briefly, all infants with two SMN2 copies achieved the primary outcome of sitting without support at the 18-month assessment, and 8 of 15 patients with three SMN2 copies met the primary endpoint of standing without support within a normal developmental window (the remaining 7 patients were younger than the age cutoff for this motor skill) [105,106]. These positive results indicated the importance of early treatment with zolgensma prior to the onset of SMA symptoms and enforced the implementation of newborn screening programs throughout the United States [107].

A phase 4 follow-up study currently monitors the long-term safety, efficacy, and durability of response to zolgensma (NCT04042025). In addition, SMART, a phase 3b, open-label, single-arm study evaluates the effect of zolgensma in pediatric patients with SMA weighing ≥8.5 kg and ≤21 kg (NCT04851873). Zolgensma is also being tested in type 2 SMA patients between 2 and 18 years in a phase 3, sham-controlled, double-blind study (NCT05089656). Finally, OFELIA, a phase 4, open-label, single-arm study, evaluates the intravenous (IV) administration of zolgensma in pediatric SMA patients from Latin America and Canada (NCT05073133).

#### 3.2.2. ASOs in SMA

##### A Splice-Switching ASO Targeting SMN2 Pre-mRNA: Nusinersen

As previously mentioned, SMN2 fails to prevent SMA. This is due to a critical C-to-T mutation at the sixth position of exon 7 [108]. Consequently, exon 7 is skipped during the splicing process of the pre-mRNA, leading to the production of SMNΔ7, a truncated and unstable protein that cannot substitute for mutant SMN1 [109].

Nusinersen, a 2′-O-MOE ASO, uses Watson–Crick pairing to specifically bind to the intronic splicing silencer-N1 (ISS-N1), a major inhibitory element in intron 7 of the SMN2 pre-mRNA. Nusinersen masks the regulatory sequences required for exon 7 splicing. As a result, it causes the incorporation of exon 7 in the SMN2 mRNA, raising the levels of functional SMN protein [110].

Propelled by the inspiring results of preclinical studies, in 2011, Ionis Pharmaceuticals began the first clinical trial of nusinersen: in a phase 1 study, single IT doses of nusinersen were administered in 28 patients with SMA type 2 and type 3 (NCT01494701, NCT01780246) [111]. This open-label study had promising results and led to the initiation of many clinical trials at multiple locations (e.g., NCT01839656) [112]. To date, two large, multicenter, randomized, sham-controlled, phase 3 clinical trials of nusinersen have been completed: ENDEAR (NCT02193074) in SMA1 and CHERISH (NCT02292537) in SMA2 patients. These studies and an open-label study in pre-symptomatic infants named NURTURE (NCT02386553) showed that nusinersen improved motor function in most treated patients [39,113,114], confirming the efficacy and tolerability of the compound. Finally, nusinersen was approved by the FDA in December 2016 to treat all types of SMA in all ages, being the first approved drug treatment for SMA [115].

Nusinersen’s EAPs were initiated in several countries following drug approval. Data published from these EAPs and analysis of seven clinical trials confirmed the therapeutic benefit of nusinersen [116]. Currently, nusinersen is being further evaluated in various clinical trials around the world: phase 3 long-term extension studies (NCT04729907, NCT02594124), studies regarding the efficacy of nusinersen in adults with SMA (NCT04576494, NCT04159987) and other studies (NCT04674618, NCT04488133, NCT04089566, NCT02386553, NCT05067790).

## 4. Duchenne Muscular Dystrophy (DMD)

### 4.1. Clinical Manifestations and Genetic Background

With a prevalence of fewer than 10 cases in 100,000 males, DMD is a debilitating X-linked neuromuscular disease [117]. It is caused by mutations in the DMD gene, which encodes dystrophin, a skeletal and cardiac muscle fiber cytoskeletal protein. Mutations abolish the formulation of the muscle isoform of dystrophin (Dp427m), resulting in progressive muscle degeneration and necrosis [118]. With optimal care offered, muscle strength deteriorates, respiratory failure and cardiomyopathy emerge, and death occurs between 20 and 40 years [119].

The field of genetic therapeutics has responded to the urgent need for a cure for this debilitating disease. Today, many drugs are approved or in late-stage clinical development that can address some of the most prevalent types of DMD. Table 3 organizes these drugs.

### 4.2. Available Gene Therapies for DMD

#### 4.2.1. GRT in DMD

In DMD, GRTs do not specifically target each mutation. Instead, they restore muscles’ function by providing either a truncated but functional DMD gene or muscle-protecting enzymes.

##### GRTs Encoding Shortened DMD Genes: Microdystrophin GRTs

As is widely known, in-frame mutations affect one or more amino acids and result in a less altered and more functional protein compared with out-of-frame mutations, which totally change the reading frame and the subsequent amino acid sequence. This is clearly seen if we compare the debilitating progress of DMD, which is caused by out-of-frame mutations in the DMD gene, with the benign clinical phenotype of Becker muscular dystrophy, which is caused by in-frame mutations in the DMD gene. In-frame mutations in Becker patients may reduce the size of the DMD gene but do not deprive its ability to encode a partially functional dystrophin protein [120].

Following a similar rationale and considering that the size of the DMD gene does not allow its transfer via an AAV vector, GRT in DMD restores the genetic defect by transferring a shortened human DMD gene, called microdystrophin. Microdystrophins are easily transferred to DMD patients via AAV vectors [117].

SRP-9001 is a micro-dystrophin GRT developed by Sarepta Therapeutics that is delivered through a rAAVrh74 vector and uses MHCK7, a muscle-specific promoter with enhanced cardiac expression. SRP-9001 was tested in Study 102, a two-part, phase 2, placebo-controlled clinical trial (NCT037691160). In the first part, SRP-9001 met the primary biological outcome of micro-dystrophin protein expression. However, it did not meet the primary functional endpoint of a statistically significant score on the North Star Ambulatory Assessment (NSAA). In the second part, patients who crossed over from the placebo group to SRP-9001 had a statistically significant difference in their NSAA compared to the score of the external cohort control group. These positive results promoted SRP-9001 to a phase 3, double-blind, placebo-controlled clinical trial called EMBARK, whose primary endpoint is the change from baseline in NSAA at the 52nd week (NCT05096221). Apart from the EMBARK study, the drug is also being evaluated in two additional trials; a phase 1/2 open-label clinical trial (NCT03375164) and a phase 1 open-label clinical trial called ENDEAVOR (NCT04626674).

PF-06939926—also known as fordadistrogene movaparvovec—is another microdystrophin GRT that uses AAV9 as a vector. Currently, its safety is being evaluated in a phase 1b, open-label clinical trial, where it is administered IV (NCT03362502). Preliminary analysis showed that PF-06939926 has an acceptable safety profile; three treatment-related serious adverse events occurred in one year (thrombocytopenia, dehydration, and acute kidney injury), and all ameliorated within 15 days. In addition, the drug proved to be potentially efficient, as it resulted in an NSAA score of (+)1 after one year, whereas the NSAA score of the external cohort control was (−)4 [121]. At the same time, the drug is currently being investigated in a phase 3 study, which primarily assesses the change from baseline in NSAA at the 52nd week (NCT04281485).

Finally, SGT-001 is a micro-dystrophin GRT developed by Solid Biosciences. It contains a neuronal nitric oxide synthase-binding domain, which acts by preventing ischemic muscular injury. SGT-001 entered clinical trials in December 2017 and is currently being evaluated in a phase 1/2 trial called IGNITE DMD, which assesses the safety and efficacy of a single IV infusion (NCT03368742).

##### A GRT Encoding the GALGT2 Enzyme: rAAVrh74.MCK.GALGT2

The effect of the microdystrophin GRT could be enhanced by a surrogate gene therapy, which does not target the genetic defect of DMD [3]. Such an approach is the AAV-mediated delivery of the gene encoding the GALGT2 enzyme. GALGT2 glycosylates α-dystroglycan in skeletal muscles, thereby increasing dystroglycan-binding proteins (one of which is dystrophin) and protecting dystrophic muscles from injury. Currently, an AAVrh74-mediated GALGT2 GRT under the control of an MCK promoter (rAAVrh74.MCK.GALGT2) is being investigated in a phase 1/2, open-label clinical trial (NCT03333590) [122].

#### 4.2.2. ASOs in DMD

Exon-skipping interventions using ASOs were the first viable precision therapies for DMD. Exon skipping relies on the use of an ASO to bind at a specific location of the DMD pre-mRNA, altering splicing to exclude the exon in the mature mRNA and, in many cases, restoring the reading frame. A major advantage of the ASOs is that most DMD mutations are concentrated between exons 43 and 53, allowing the application of ASOs that target this region to a large patient population.

##### A Splice-Switching ASO Targeting Exon 51 of the DMD Pre-mRNA: Eteplirsen

The first approved drug for DMD was eteplirsen, a PMO developed by Sarepta Therapeutics. Eteplirsen binds to the complementary exon 51 of the DMD pre-mRNA and causes its skipping during the mRNA splicing process. The absence of exon 51 in the mature DMD mRNA restores the reading frame and results in a functional, slightly shortened dystrophin protein. Despite its very specific action, eteplirsen applies to a wide range of patients, as 13–14% of all DMD cases have mutations that can be addressed by skipping exon 51, such as deletion of exons 49–50 or 52–63 [123].

Eteplirsen received accelerated FDA approval in September 2016. Its safety and biochemical efficacy were proven in an open-label, phase 2 dose-escalation study and showed its potential to become a disease-modifying drug for DMD (NCT00844597) [124]. In a 12-participant clinical trial that began in July 2011, eteplirsen did not show any serious adverse effects and restored dystrophin levels, as assessed by the percentage of dystrophin-positive muscle fibers during immunohistochemistry of muscle biopsies (NCT01396239) [125]. At the request of the FDA, due to the small number of participants, Sarepta Therapeutics provided more data (Western blot from other biopsies of eteplirsen-treated patients) from its phase 3 confirmation trial, PROMOVI (NCT02255552) [40]. Overall, the levels of dystrophin restoration were not proven significant. Still, they were enough to confirm the mechanism of action of eteplirsen. Considering this and the proven safety of eteplirsen, the FDA finally approved the ASO [126]. However, it was mentioned that eteplirsen’s clinical benefits are yet to be established, and the conduction of confirmatory trials was required. The longitudinal effect of eteplirsen versus historical control on ambulation in DMD was confirmed by an open-label study of the ASO in subjects who participated in the study NCT01396239. Over 3 years of follow-up, eteplirsen-treated patients assessed by 6MWT showed a slower rate of decline in ambulation than untreated matched historical controls [127]. Currently, Sarepta Therapeutics has three ongoing phase 2 (NCT04179409, NCT03985878) and phase 3 (NCT03992430) clinical trials to evaluate the safety and efficacy of eteplirsen at multiple centers around the world.

##### A Splice-Switching ASO Targeting Exon 53 of the DMD Pre-mRNA: Golodirsen

Golodirsen is another ASO from Sarepta Therapeutics’ PMO platform approved under accelerated review for the treatment of DMD in patients with mutations amenable to exon 53 skipping (approximately 7.7% of all DMD mutations). In a two-part, phase 1/2 clinical trial of golodirsen, increased dystrophin production was observed in the skeletal muscles of treated patients, indicating a potential clinical benefit (a minimum of 48 weeks of treatment with the ASO resulted in an increase in the dystrophin levels from 0.10% of normal, at baseline, to 1.02% of normal) (NCT02310906) [128]. Among the non-severe adverse effects reported were headache, pyrexia, cough, nasopharyngitis, GI symptoms, and hypersensitivity reactions. In addition, because some animals who received golodirsen presented renal toxicity, including potentially fatal glomerulonephritis, FDA required renal function monitoring in patients treated with the ASO [41].

##### A Splice-Switching ASO Targeting Exon 45 of the DMD Pre-mRNA: Casimersen

Casimersen is a PMO by Sarepta Therapeutics that induces the skipping of exon 45 in the dystrophin gene. It is applied to DMD patients with mutations in exon 45, who comprise 8.1% of all DMD patients. It received accelerated approval by FDA in February 2021, based on an increase in dystrophin production from a double-blind, placebo-controlled study (NCT02500381). As with golodirsen, renal toxicity observed in nonclinical studies indicated the monitoring of renal function in patients taking casimersen [42].

Because further confirmatory studies were required to verify and describe the anticipated clinical benefits of golodirsen and casimersen, Sarepta Therapeutics is conducting an ongoing, double-blind, placebo-controlled, multicenter study (NCT02500381) and a phase 3, long-term, OLE study (NCT03532542).

##### A Splice-Switching ASO Targeting Exon 53 of the DMD Pre-mRNA: Viltolarsen

Viltolarsen is another PMO that treats DMD patients with exon 53 mutations. It was developed by Nippon Shinyaku in collaboration with the National Center of Neurology and Psychiatry. Based on the presence of dystrophin restoration (without measurement of muscle function) and the absence of serious adverse effects in two-phase 1/2 and phase 2 clinical studies with a total of 32 patients (NCT02740972, JapicCTI-163291) [129,130], viltolarsen received accelerated approval from the FDA in August 2020 [44]. However, for similar reasons to the two previous ASOs, renal function has to be also monitored in the case of viltolarsen [131].

As part of the accelerated approval process, the FDA required the company to conduct a clinical trial to confirm the clinical benefit of the ASO.

An interim analysis of the results of the OLE trial of NCT02740972 showed that viltolarsen-treated patients showed a statistically significant benefit regarding the primary endpoint of “time to stand” and additional secondary endpoints of motor function compared to DMD matched historical controls (NCT03167255) [132]. Except for the above trial, there are other phase 2, phase 3, and phase 4 clinical trials evaluating the safety and efficacy of viltolarsen worldwide, mainly organized by NS Pharma (NCT04956289, NCT04768062, NCT04060199, NCT04687020, NCT05135663).

##### A Peptide-Conjugated, Splice-Switching ASO Targeting Exon 51 of the DMD Pre-mRNA: SRP-5051

On the downside, PMOs face a major challenge in cellular uptake; because of their nonionic backbone, they have difficulty diffusing across cellular membranes, especially in the heart [133]. Therefore, numerous approaches have been designed to improve PMO delivery. One of them is peptide PMO (PPMO) technology [134].

Known as next-generation eteplirsen, SRP-5051 is a PPMO ASO that triggers exon 51 skipping in the DMD pre-mRNA, developed by Sarepta Therapeutics. SRP-5051 employs an innovative delivery system called cell-penetrating peptides (CPPs). CPPs are short peptide ligands that most cell types can take up. As a result, they allow the enhanced delivery of conjugated biomolecules, such as ASOs, into tissue cells, improving their pharmacological properties [135].

Based on the promising results of SRP-5051 in preclinical studies, the ASO has been advanced to clinical trials [136]. Sarepta Therapeutics has an ongoing, phase 2, two-part, multiple-ascending-dose study of SRP-5051, named MOMENTUM (NCT04004065). In part A of the trial, which evaluated the safety and tolerability of the ASO at multiple-ascending dose levels, patients received monthly IV infusions of SRP-5051, starting at 4 mg/kg and increasing to 40 mg/kg. On 7 December 2020, Sarepta Therapeutics announced the results from part A. Patients treated with SRP-5051, 20 mg/kg monthly, for 12 weeks, had better results when compared to DMD patients treated with eteplirsen, 30 mg/kg weekly, for 24 weeks (during the PROMOVI trial). More specifically, SRP-5051-treated patients showed higher concentration of the ASO in their muscles, increased exon skipping, and higher dystrophin levels. These early positive results demonstrate the potential efficacy of SRP-5051. Dose escalation is currently ongoing in part B of the MOMENTUM trial [137].

##### A Splice-Switching ASO Targeting Exon 45 of the DMD Pre-mRNA: Renadirsen

In continuation of exon-skipping treatments, Daiichi Sankyo developed renadirsen, an ASO that promotes exon 45 skipping. Renadirsen’s therapeutic properties are not limited to DMD skeletal muscle symptoms but also target other symptoms, such as decreased heart muscle function [138]. The ASO is modified with 2′-O-methyl RNA/ENA (2′-O,4′-C-ethylene-bridged nucleic acid) chimera PS, a novel chemistry design that enhances the nuclease resistance and the affinity toward complementary RNA strands. In preclinical studies, renadirsen demonstrated substantial dystrophin restoration via exon 45 skipping, suppression of Ca^2+^ overflow, and secretion of creatine kinase in myotube cells derived from patients’ iPS cells [138]. In October 2015, renadirsen was advanced into an open-label, phase 1/2 clinical trial, which evaluated the safety and efficacy of the ASO (NCT02667483). The trial results were announced in January 2021; renadirsen was evaluated as safe, not causing any serious adverse effects. Regarding the trial’s primary endpoint, an increase in dystrophin protein levels was observed in several patients. In addition, the production of mRNA with skipping of exon 45 (the trial’s secondary endpoint) was found in all patients [139]. Currently, renadirsen is being tested in a phase 2, long-term extension study (NCT04433234). The trial consists of DMD patients who have completed the previous phase 1/2 trial and aims to evaluate the long-term safety and efficacy of the ASO.

##### A Splice-Switching Stereopure ASO Targeting Exon 51 of the DMD Pre-mRNA: Suvodirsen

Suvodirsen was a splice-switching, stereopure ASO developed by Wave Life Sciences that induced the skipping of exon 51 in DMD pre-mRNA. Results from preclinical studies of suvodirsen in cell cultures were promising, indicating enhanced exon skipping and restoration of dystrophin protein levels compared with other exon-51-skipping ASOs. As a result, suvodirsen was advanced in clinical trials in November 2017. In March 2019, the ASO completed a double-blind, placebo-controlled, phase 1 clinical trial which proved the safety of IV, single-ascending doses of suvodirsen (NCT03508947) [140] and was advanced to a phase 1 OLE study. Although there were no safety concerns, interim results from the OLE study showed no change from baseline in dystrophin expression. As a result, in December 2019, Wave Life Sciences announced the discontinuation of the two ongoing suvodirsen trials, the OLE study and another phase 2/3 trial (NCT03907072) [141].

##### A Splice-Switching ASO Targeting Exon 53 of the DMD Pre-mRNA: WVE-N531

WVE-N531, by Wave Life Sciences, is another splice-switching ASO that causes the skipping of exon 53 during DMD pre-mRNA processing. Its uniqueness lies in the fact that it is Wave’s first exon-skipping candidate to use its novel PN backbone chemistry modifications (PN chemistry). Preclinical studies of WVE-N531 appeared very promising regarding the dose-dependent increase in dystrophin production, survival at 40 weeks, intracellular access, and distribution of the ASO compared to Wave’s first-generation ASOs. WVE-N531 entered clinical development in March 2021. Currently, it is being evaluated in a phase 1b/2a clinical trial, which primarily focuses on the safety and tolerability of ascending doses of the ASO in DMD boys (NCT04906460). The first dosing of WVE-N531 was initiated in September 2021. More clinical data, which will enable decision-making on the next steps for WVE-N531, are expected to be generated through 2022 [142].

##### A Splice-Switching ASO Targeting Exon 44 of the DMD Pre-mRNA: NS-089/NCNP-02

Apart from viltolarsen, Nippon Shinyaku, together with the National Center of Neurology and Psychiatry of Japan, has developed another PMO to treat DMD. The ASO is named NS-089/NCNP-02 and acts through exon 44 skipping.

Currently, NS-089/NCNP-02 is being tested in an exploratory study with six participants, aiming to assess the safety, tolerability, efficacy, and pharmacokinetics of NS-089/NCNP-02 and determine the dosage for subsequent studies (NCT04129294).

##### An snRNP-Incorporated, Splice-Switching ASO Targeting Exon 2 of the DMD Pre-mRNA: scAAV9.U7.ACCA

ASOs may be widely used in gene therapy of DMD, but this does not mean that they do not have any disadvantages. ASOs are known to stimulate immune response and to be sensitive to degradation. Consequently, an approach that overcomes these difficulties is the incorporation of the ASO in a U7 snRNP molecule. U7 snRNP is a uridine-rich small nuclear ribonucleoprotein (a complex composed of small nuclear RNA and proteins) that, when modified, manipulates the splicing process of DMD mRNA and can be used therapeutically in DMD. Importantly, this molecule has minimal size, concentrates in the nucleus, and does not induce an immune reaction [143].

This approach is currently applicable to treating DMD patients with exon 2 duplication. An ASO that causes the skipping of the additional exon 2 in the premature DMD mRNA is incorporated in U7 snRNP. The therapeutic complex is delivered to patients through a scAAV9 vector. The resulting mature mRNA can be wild-type mRNA (contains one copy of exon 2 and results in asymptomatic patients) or Del2 mRNA (contains no copy of exon 2 and results in patients being able to walk even at 80 years of age) [3]. This promising agent is currently being tested in a phase 1/2 open-label clinical trial aiming to assess its safety and obtain preliminary efficacy data (NCT04240314).

## 5. Limb-Girdle Muscular Dystrophy (LGMD)

### 5.1. Clinical Manifestations and Genetic Background

First defined by Walton and Nattrass in 1954, LGMDs are a group of rare, autosomal inherited muscular dystrophies of late childhood to adult-onset, with a prevalence in about 1–9 in every 100,000 individuals [144]. As the name indicates, LGMDs are primarily characterized by worsening symmetrical atrophy of the proximal limb muscles [145]. However, the disease is rarely limited to these sites; it often extends to the distal skeletal, cardiac, and respiratory muscles. As a result, symptoms frequently occur, such as muscle cramps, dilated cardiomyopathy, arrhythmias, and respiratory dysfunction [146,147,148,149]. The progression of these symptoms varies considerably between the various LGMD subtypes due to the different genetic causes of each disease. The problem generally stems from the defective protein synthesis in muscle fibers in locations such as the nucleus, the sarcoplasm, and the extracellular matrix [150,151,152].

Nowadays, more and more emphasis is placed on distinguishing the pathogenic genetic variants of LGMD. It is of note that, although the clinical characteristics of each subtype long guided the diagnosis of the LGMDs, this changed in 2018; during the 229th ENMC International Workshop, the European Neuromuscular Centre proposed a new nomenclature that integrates the use of genetics for its classification [153].

Similarly, therapeutic interest has shifted to gene therapies. While pharmacological approaches have failed to inhibit the course of the disease, disease-modifying gene therapies seem to be the ideal way to target monogenetic, recessive diseases with a clear genetic cause [122,154].

The LGMDs with the highest prevalence are LGMD type 2A (calpainopathy), LGMD type 2B (dysferlinopathy), LGMD type 2D (α-sarcoglycan), and LGMD type 2E (β-sarcoglycan). Meanwhile, gene therapies in clinical trials are currently available for LGMD type 2E and LGMD type 2I; these therapies are presented in Table 4.

### 5.2. Available Gene Therapies for LGMD

#### 5.2.1. GRT in LGMD

##### A Dual-Vector GRT for LGMD Type 2B Dysferlinopathy: AAV.DYSF.DV

Dysferlin (dystrophy-associated fer-1-like protein) is a transmembrane protein of the skeletal muscles. It is mainly associated with the sarcolemma repair process and is particularly involved in the calcium-mediated fusion of vesicles with the plasma membrane. Dysferlin deficiency causes dystrophic phenotypes, the most common of which are LGMD type 2B [155], Miyoshi myopathy [156], and distal myopathy with anterior tibialis onset [157]. All of these subtypes show remarkable pathological similarity, and, as the disease progresses, phenotypical profiles overlap significantly, resulting in a common, “proximal-distal limb-girdle phenotype” [158,159,160,161,162,163].

The lack of dysferlin that causes all these disorders is due to mutations in the DYSF gene, located on chromosome 2p13 [164]. Thus, repairing this gene defect with GRT seems to be the ideal approach, as it will improve all dysferlin-deficient pathological phenotypes. The only limitation in this approach is the huge size of the DYSF gene (it is composed of 55 exons that span 233.140 bp of genomic DNA and gives rise to a 6.9-kb-wide transcript), which far exceeds the 5 kb packaging capacity of AAV vectors [164].

Thus, Sondergaard’s group developed a dual-vector GRT named AAV.DYSF.DV. Two different segments from dysferlin cDNA were introduced into two AAV serotype rh74 vectors. These segments show homology in an overlap region of 1kb. Thus, when introduced in vivo, they facilitate the production of full-length dysferlin via homologous recombination [165].

In preclinical studies in mice and nonhuman primates, treatment has been shown to be safe and effective. In the short term, dysferlin expression and membrane-repair function were observed, along with muscle recovery. In the long term, continuous dysferlin expression was observed after 12–18 months [166].

Positive preclinical results of AAV.DYSF.DV paved the way for the clinical validation of the GRT. In July 2019, a phase 1 preliminary human study that validated intramuscular (IM) delivery of AAV.DYSF.DV to small forearm muscles of two LGMD type 2B patients was completed (NCT02710500). Results have not been posted yet, but the treatment did not show any significant safety concerns [161].

##### A Human γ-Sarcoglycan GRT for LGMD2C Sarcoglycanopathy

Among the proteins that structurally stabilize sarcolemma is the sarcoglycan complex, which is part of the aforementioned dystrophin-associated glycoprotein complex. The sarcoglycan complex consists of six transmembrane proteins (α-, β-, γ-, δ-, ε-, ζ-sarcoglycan) closely interdependent to the extent that the deficiency of one protein destabilizes the whole complex [167,168,169]. Such a deficiency occurs when the genes encoding α-, β-, γ-, and δ-sarcoglycans undergo mutations, causing the four autosomal recessive LGMDs (2D, 2E, 2C, and 2F), also known as sarcoglycanopathies (10–25% of LGMD cases) [170]. Sarcoglycanopathies usually first appear in childhood and follow a rapidly deteriorating course, with loss of ambulation occurring in the second decade of life [170].

LGMD type 2C sarcoglycanopathy is characterized by γ-sarcoglycan deficiency due to mutations in the γ-sarcoglycan gene. In 2012, Herson et al. reported one of the first GRT clinical trials in this LGMD type. Their phase 1 study involved nine non-ambulatory patients with a homozygous mutation of the γ-sarcoglycan gene and no γ-sarcoglycan immunostaining on muscle biopsy (NCT01344798) [171]. In three patients, an AAV1-mediated GRT encoding the human γ-sarcoglycan gene was administered IM in three escalating doses. No serious adverse effects occurred during 6 months of follow-up. Immunohistochemical analysis of muscle biopsy specimens performed 30 days later showed γ-sarcoglycan expression in all three patients who received the highest dose. At the same time, a real-time polymerase chain reaction detected γ-sarcoglycan mRNA. In one patient, the γ-sarcoglycan protein was detected by Western blot. For two other patients who received the low and intermediate doses, discrete levels of γ-sarcoglycan expression were also detectable. They concluded that the GRT is safe and can effectively induce the expression of the γ-sarcoglycan protein in the muscles.

##### A Human hSGCA GRT for LGMD2D Sarcoglycanopathy: rAAV1.tMCK.hSGCA

LGMD type 2D is the most common form of sarcoglycanopathy and is due to α-sarcoglycan deficiency [172]. To restore α-sarcoglycan, Mendell’s group used a rAAV to transfer the full-length human hSGCA cDNA under the control of a muscle-specific creatine kinase promoter (tMCK) [173]. When the GRT was tested via IM injection in mice, it proved to be non-toxic and restored α-sarcoglycan expression. Thus, in March 2008, rAAV1.tMCK.hSGCA entered a phase 1, double-blind clinical trial with six participants (NCT00494195). GRT was administered IM in the one extremity of the patients, while simple saline was administered to the other extremity. The treatment did not trigger an immune response, fulfilling the study’s primary endpoint. In addition, it proved to be effective, as a large expression of the hSGCA gene was observed in five of six subjects in muscle biopsies conducted after 6 weeks (two subjects), 12 weeks (one subject), and 6 months (three subjects) [173,174].

##### A Human hSGCA GRT for LGMD2D Sarcoglycanopathy: AAVrh74.tMCK.hSGCA

Following the promising results of the previous study, GRT using hSGCA and tMCK proceeded to a phase 1/2a, dose-escalation, open-label clinical trial with six participants. In this trial, however, the genetic cargo was transferred through a scAAVrh74 vector, whose benefits had been demonstrated by clinical trials for DMD [175]. This GRT by Sarepta Therapeutics, also named MYO-201, was intravascularly administered to patients via a major lower limb artery of each leg sequentially by isolated limb infusion (ILI) (NCT01976091). Inspired by malignant salvage chemotherapy, ILI specifically targeted the pathological area, requiring less viral therapeutic load. As confirmed by muscle biopsies, the ILI-delivered GRT increased the expression of α-sarcoglycan when administered at a low dose. In addition, functional improvement was observed in the targeted limb muscles. Though, proximal muscles responsible for major functions, such as ambulance, remained weakened. Thus, it is predicted that future trials will target the proximal muscles through systematic gene delivery [176].

##### A Human SGCB GRT for LGMD Type 2E Sarcoglycanopathy: SRP-9003

LGMD type 2E, also known as beta-sarcoglycanopathy, is caused by a mutation in the β-sarcoglycan (SGCB) gene and is one of the most severe forms of LGMD. It causes significant disability in children and frequently leads to early mortality due to pulmonary or cardiac complications [177,178].

A promising therapeutic agent for this disease’s skeletal and cardiac muscle deficits is a GRT called SRP-9003 by Sarepta Therapeutics. SRP-9003 uses a scAAVrh74 vector to deliver a normal SGCB gene, driven by a muscle-specific promoter [179]. Based on optimistic preclinical indications, SRP-9003 was advanced, in October 2018, in a phase 1/2 open-label clinical trial, which is currently ongoing (NCT03652259). The trial assesses the safety, tolerability, and efficacy of IV administered SRP-9003. In March 2021, results from the study were presented: 2 years after a single administration of SRP-9003, beta-sarcoglycan protein expression levels were significantly sustained (in the low-dose cohort, mean beta-sarcoglycan expression was 54% at 24 months, compared to 36% at day 60) and functional outcomes measured were meaningfully strong. In addition to beta-sarcoglycan, strong expression of delta-sarcoglycan and gamma-sarcoglycan proteins was observed in the high-dose cohort, indicating that SRP-9003 may also restore the dystrophin-associated protein complex [180].

##### A Human FKRP GRT for LGMD Type 2I: LION-101

LGMD type 2I is caused by an autosomal recessive mutation in the fukutin-related protein (FKRP) gene. FKRP gene encodes a ribitol-5-phosphatase transferase necessary for glycosylation of alpha-dystroglycan, thereby the muscle fiber’s matrix-anchoring [181]. Often described as having DMD-like phenotypes, patients with LGMD2I develop symptoms at a median age of 11.5 years [182]. Their motor function deteriorates as they become older, and 23–26 years later, they are confined to a wheelchair.

Seeking to meet the need for treatment for LGMD type 2I, an IV administered GRT by Asklepios BioPharmaceutical, named LION-101, uses a rAAV to transfer a physiological copy of the human FKRP gene. After its efficacy and tolerability were confirmed in preclinical models, LION-101 was recently advanced to a phase 1/2, double-blind, placebo-controlled, dose-escalation study, which evaluates the safety of the GRT in genetically confirmed LGMD2I patients (NCT05230459).

##### A Human FKRP GRT for LGMD Type 2I: GNT0006

Another AAV-delivered FKRP GRT, named GNT0006, comprises an additional potential treatment for LGMD type 2I. In February 2022, a clinical trial of GNT0006, sponsored by Atamyo Therapeutics, was initiated and is currently ongoing. This phase 1/2 study primarily assesses the safety, pharmacodynamics, efficacy, and immunogenicity of the drug and consists of two parts: an open-label, dose-escalation phase, and a double-blind, placebo-controlled phase, both with a long-term follow-up period (NCT05224505).

## 6. Myotonic Dystrophy (DM)

### 6.1. Clinical Manifestations and Genetic Background

Myotonic dystrophy type 1 (DM1) and type 2 (DM2) are multisystemic autosomal dominant muscular disorders caused by RNA-mediated toxicity due to a tandem repeat expansion [183]. Due to having a similar underlying mechanism of RNA-caused toxicity, both DM subtypes share common clinical features. However, the genetic therapeutic approach differs between the two genetically different DM subtypes. In this review, we focus only on the genetic treatment for DM1, as most preclinical and clinical studies were done on DM1 models and patients.

The genetic cause of DM1 is a CTG trinucleotide expansion in the 3′ untranslated region (UTR) of the dystrophia myotonic protein kinase (DMPK) gene. The DMPK gene codes for a serine-threonine kinase mainly expressed in smooth, skeletal, and cardiac muscles [183,184,185,186]. Transcription of mutated DMPK generates mRNAs with long CUG repeats, which act in many different ways. They mainly lead to defects in the splicing of numerous pre-mRNAs by trapping RNA-binding proteins [187,188,189,190,191]. Splicing defects of the muscle chloride channel, the insulin receptor, and cardiac troponin T transcripts lead to myotonia, insulin resistance, and cardiac abnormalities, respectively [192,193,194]. In addition, mutated DMPK transcripts modify the expression of various transcription factors and miRNAs, altering gene expression or interacting with the translation machinery, causing the production of potentially toxic proteins [195,196,197].

All the above pathogenetic mechanisms lead to the clinical features of DM1. The disease’s main symptom is myotonia, which was mentioned above, along with progressive muscle wasting and weakness of cranial, trunk, and distal limb muscles [197]. All types of DM1 are characterized by a high disease burden and may lead to premature death.

Gene therapies for myotonic dystrophy studied in clinical trials focus on destroying mutated DMPK mRNAs. At present, only one siRNA therapy by Avidity Biosciences, AOC1001, is being tested in clinical trials (see Table 5).

### 6.2. Available Gene Therapies for DM

#### 6.2.1. ASOs in DM

##### An ASO RNase H1-Mediated Inhibitor of DMPK mRNA: IONIS-DMPKRx

IONIS-DMPKRx by Ionis Pharmaceuticals was an ASO that base-paired with a specific 3′-UTR sequence outside the repeat tract and induced RNase H-mediated degradation of DMPK mRNA. It was selected among 3.000 other candidate drugs and, after yielding positive results in the preclinical test, proceeded to a phase 1/2a placebo-controlled clinical trial (NCT02312011) [198,199]. The ASO was found to be safe, even in its highest dose. However, the amount of treatment that eventually reached the tissues was insufficient to elicit the desired therapeutic effects. Thus, Ionis stopped the development of the ASO and focused on drugs with advanced delivery properties, which used ligand-conjugated antisense technology [200].

#### 6.2.2. RNAi in DM

##### A siRNA Inhibitor of DMPK mRNA: AOC-1001

AOC-1001—a member of Avidity Biosciences’ platform called Antibody Oligonucleotide Conjugates (AOCs)—constitutes a pioneer drug in terms of delivery. AOC-1001 targets DMPK mRNA with a siRNA conjugated with a proprietary monoclonal antibody, which binds to the transferrin receptor 1. Preclinical studies have demonstrated the safety and the efficacy of the drug in a durable, dose-dependent reduction of DMPK RNA across a wide range of muscle cells. Thus, in August 2021, the advancement of the drug in a phase 1/2 clinical study was announced [201], and, in November 2021, the drug was administered to the first participants, marking the first time that an AOC was administered to patients (NCT05027269) [202]. The trial assesses the safety and tolerability of single-ascending and multiple-ascending doses of IV administered AOC-1001. In parallel, it explores the clinical activity of AOC-001 by measuring mobility and muscle strength, patient-reported outcomes, and quality of life. As soon as the post-treatment period ends, patients can choose to enroll in an OLE study.

## 7. Conclusions and Perspectives

In summary, gene therapy represents the most promising and revolutionary therapeutic approach for neuromuscular diseases. However, the implementation of this technology is still in the early stages, and some limitations need to be addressed.

Toxicity related to the gene therapy’s on- and off-target effects displays a major limitation, despite the fact that (a) the nervous system is generally well protected from inflammatory response, and (b) the administration of AAV in human CNS has been proven safe. Regarding on-target effects, the unknown number of transgenes transferred in the cell may lead to excessive correction of the gene defect (over- or under-expression of the healthy allele) and potential long-term neurotoxicity [203]. In terms of off-target effects, gene-specific therapies can trigger immune responses in cell types that should not be targeted. For example, the IT administration of zolgensma in non-human primates triggered dorsal root ganglia inflammation [204,205]. In addition, there is evidence that the AAV genome triggers an innate immune reaction; however, the mechanism has not been fully elucidated [206]. Moreover, in the case of utilizing high doses of viral vectors, the risks of insertional mutagenesis and genotoxicity should be considered [207].

Another significant aspect refers to limited drug efficacy. This is due to the genetic agents’ low bioavailability and cell selectivity in many cases. Cardiomyopathy in DMD constitutes a characteristic example, as ASOs do not efficiently target the skeletal and cardiac muscle [208]. Similarly, in the case of ALS, pharmacological agents used in the context of gene therapy do not efficiently reach the cerebral cortex’s protected regions and the spinal cord. To overcome this and achieve the maximum tissue targeting, the drug administration needs to occur IT. This is not only technically challenging and expensive but also emotionally draining for the patient. Limited drug efficacy can also be attributed to the degradation of drugs such as ASOs by endo- and exo-nucleases. This requires a drug re-administration, usually IT, to ensure an effective dose. Additionally, many patients have been naturally infected by wild-type AAV and have developed neutralizing antibodies against rAAV capsids, which block rAAV gene delivery [209,210]. The immune-privileged nervous system can tolerate a high neutralizing antibodies titer following a direct CNS delivery but is not completely spared from circulating neutralizing antibodies [211,212].

The above limitations may be challenging, but gene therapies are being modified incessantly, and many innovative technologies are currently under development to enhance their target potency and improve their safety. The expression levels of the gene product should be thoroughly estimated to ensure long-term safety. In that sense, future gene therapies should use tightly regulated endogenous promoters and allow for inducible regulation of the transferred gene to minimize adverse events. Moreover, to enhance the uptake of gene therapies by tissues and prevent their nuclease-mediated degradation, research should focus on finding new chemical modifications and appropriate conjugates that enable bioavailability. Moving on to the clinical application of gene therapies, the drugs should ideally be tested in clinical trials on genetically homogenous populations. Such populations can now be identified more efficiently as new genomic sequencing techniques emerge and genetic screening becomes more and more applicable. There is also a need to identify diagnostic and prognostic biomarkers that will diagnose the disease in its early stages and help predict the clinical outcome. Biomarkers could also function as surrogates for clinical endpoints, as they properly indicate the course of the disease. Lastly, a deeper molecular insight into the disease-causing factors will facilitate the development of better pre-clinical models, leading to more accurate predictions regarding the clinical outcome.

In conclusion, despite the caveats discussed herein, gene therapies targeting the nervous system represent the most promising therapeutic approach, with remarkable progress achieved during the last decade. This is illustrated by the numerous approvals of genetic treatments for motor neuron diseases, muscular dystrophies, and additional nervous system disorders not discussed in this review. Gene therapies have altered the therapeutic landscape for an array of devastating neurological diseases associated with an adverse clinical outcome until recently. Based on the latest successful efforts, there is currently a large pipeline of genetic drugs in clinical development. In our opinion, the close interaction between experimental and clinical research and the significant experience gained from exploratory clinical trials until now can ensure a successful pathway towards developing novel and effective gene therapies targeting motor neuron diseases and muscular dystrophies.

## Figures and Tables

**Table 1:** Gene-specific therapies for ALS tested in clinical trials.

	Drug	Sponsor/Collaborators	Target of Drug	Status	NCT Number	Study Completion
Gene replacement therapies (GRTs)						
1	Engensis	Helixmith	GRT of HGF gene	Phase 2	NCT05176093	November 2022
2	Engensis	Helixmith	GRT of HGF gene	Phase 2	NCT04632225	July 2022
Antisense oligonucleotides (ASOs)						
1	BIIB078	Biogen	ASO that targets C9ORF72 mRNA	Phase 1	NCT04288856	July 2023
2	BIIB105	Ionis/Biogen	ASO that targets ATXN2 mRNA	Phase 1	NCT04494256	December 2024
3	WVE-004	Wave Life Sciences	ASO that targets C9orf72 mRNA	Phase 1/2	NCT04931862	February 2023
4	Jacifusen	Ionis	ASO that targets FUS mRNA	Phase 3	NCT04768972	March 2024
5	Tofersen	Ionis/Biogen	ASO that targets SOD1 mRNA	Phase 3	NCT03070119	June 2024
6	Tofersen	Biogen	ASO that targets SOD1 mRNA	Phase 3	NCT04856982	August 2027

**Table 2:** Gene-specific therapies for SMA tested in clinical trials.

	Drug	Sponsor/Collaborators	Target of Drug	Status	NCT Number	Study Completion
Gene replacement therapies (GRTs)						
1	Zolgensma	Novartis	AAV-based GRT of SMN gene	Phase 3	NCT04851873	August 2023
2	Zolgensma	Novartis	AAV-based GRT of SMN gene	Phase 3	NCT05089656	October 2024
3	Zolgensma	Novartis	AAV-based GRT of SMN gene	Phase 4	NCT05073133	May 2023
4	Zolgensma	Novartis	AAV-based GRT of SMN gene	Phase 4	NCT04042025	December 2035
Antisense oligonucleotides (ASOs)						
1	Nusinersen	Biogen	ASO that targets SMN2 mRNA	Phase 2	NCT02386553	January 2025
2	Nusinersen	Biogen	ASO that targets SMN2 mRNA	Phase 2/3	NCT04089566	July 2023
3	Nusinersen	Biogen	ASO that targets SMN2 mRNA	Phase 3	NCT02594124	August 2023
4	Nusinersen	Biogen	ASO that targets SMN2 mRNA	Phase 3	NCT04729907	May 2026
5	Nusinersen	Biogen	ASO that targets SMN2 mRNA	Phase 3	NCT05067790	June 2027
6	Nusinersen	Biogen	ASO that targets SMN2 mRNA	Phase 4	NCT04488133	September 2024
7	Nusinersen	CHU de Reims	ASO that targets SMN2 mRNA	NA	NCT04576494	May 2024
8	Nusinersen	CHU de Nice	ASO that targets SMN2 mRNA	NA	NCT04159987	November 2022
9	Nusinersen	Policlinico Gemelli	ASO that targets SMN2 mRNA	NA	NCT04674618	December 2022

**Table 3:** Gene-specific therapies for DMD tested in clinical trials.

	Drug	Sponsor/Collaborators	Target of Gene Therapy	Status	NCT Number	Study Completion
Gene replacement therapies (GRTs)						
1	SRP-9001	Sarepta Therapeutics	GRT of micro-dystrophin gene	Phase 1	NCT04626674	July 2026
2	PF-06939926	Pfizer	GRT of micro-dystrophin gene	Phase 1	NCT03362502	May 2028
3	rAAVrh74.MCK.GALGT2	Nationwide Children’s Hospital	GRT of GALGT2 gene	Phase 1/2	NCT03333590	November 2021
4	SRP-9001	Sarepta Therapeutics	GRT of micro-dystrophin gene	Phase 1/2	NCT03375164	April 2023
5	SGT-001	Solid Biosciences	GRT of micro-dystrophin gene	Phase 1/2	NCT03368742	December 2028
6	SRP-9001	Sarepta Therapeutics	GRT of micro-dystrophin gene	Phase 3	NCT05096221	November 2024
7	PF-06939926	Pfizer	GRT of micro-dystrophin gene	Phase 3	NCT04281485	September 2028
Antisense oligonucleotides (ASOs)						
1	NS-089/NCNP-02	Nippon Shinyaku	ASO that targets exon 44 of DMD mRNA	Phase 1/2	NCT04129294	December 2021
2	WVE-N531	Wave Life Sciences	ASO that targets exon 53 of DMD mRNA	Phase 1/2	NCT04906460	September 2022
3	scAAV9.U7.ACCA	Megan Waldrop/Audentes Therapeutics	snRNA that targets exon 2 of DMD mRNA	Phase 1/2	NCT04240314	November 2025
4	Eteplirsen	Sarepta Therapeutics	ASO that targets exon 51 of DMD mRNA	Phase 2	NCT04179409	September 2022
5	DS-5141b	Daiichi Sankyo (Japan)	ASO that targets exon 45 of DMD mRNA	Phase 2	NCT04433234	March 2023
6	Viltolarsen	NS Pharma	ASO that targets exon 53 of DMD mRNA	Phase 2	NCT05135663	July 2023
7	Vitolarsen	NS Pharma	ASO that targets exon 53 of DMD mRNA	Phase 2	NCT04956289	May 2024
8	SRP-5051	Sarepta Therapeutics	ASO that targets exon 51 of DMD mRNA	Phase 2	NCT04004065	August 2024
9	Eteplirsen	Sarepta Therapeutics	ASO that targets exon 51 of DMD mRNA	Phase 2	NCT03985878	February 2027
10	Casimersen & Golodirsen	Sarepta Therapeutics	ASOs that target exon 45 & exon 53 of DMD mRNA	Phase 3	NCT02500381	April 2024
11	Vitolarsen	NS Pharma	ASO that targets exon 53 of DMD mRNA	Phase 3	NCT04060199	December 2024
12	Eteplirsen	Sarepta Therapeutics	ASO that targets exon 51 of DMD mRNA	Phase 3	NCT03992430	February 2026
13	Vitolarsen	NS Pharma	ASO that targets exon 53 of DMD mRNA	Phase 3	NCT04768062	June 2026
14	Casimersen & Golodirsen	Sarepta Therapeutics	ASOs that target exon 45 & exon 53 of DMD mRNA	Phase 3	NCT03532542	August 2026
15	Vitolarsen	NS Pharma	ASO that targets exon 53 of DMD mRNA	Phase 4	NCT04687020	November 2031

**Table 4:** Gene-specific therapies for LGMD tested in clinical trials.

	Drug	Sponsor/Collaborators	Target of Gene Therapy	Status	NCT Number	Study Completion
1	SRP-9003	Sarepta Therapeutics	GRT of SGCB gene	Phase 1/2	NCT03652259	February 2025
3	LION-101	Asklepios Biopharmaceutical	GRT of FKRP gene	Phase 1/2	NCT05230459	December 2028
2	GNT0006	Atamyo Therapeutics	GRT of FKRP gene	Phase 1/2	NCT05224505	October 2030

**Table 5:** Gene-specific therapies for DM tested in clinical trials.

	Drug	Sponsor/Collaborators	Target of Gene Therapy	Status	NCT Number	Study Completion
1	AOC 1001	Avidity Biosciences	siRNA that targets DMPK mRNA, conjugated with a monoclonal antibody that binds to TfR1	Phase 1/2	NCT05027269	September 2023

## Data Availability

Not applicable.

## Abbreviations

AAV	adeno-associated virus
AGO2	argonaute 2
AIDS	acquired immunodeficiency syndrome
ALS	amyotrophic lateral sclerosis
ASO	antisense oligonucleotide
ATXN2	ataxin-2
BBB	blood–brain barrier
Cas	CRISP-associated
CNS	central nervous system
CPP	cell-penetrating peptide
CSF	cerebrospinal fluid
C9orf72	chromosome 9 open reading frame 72
DM	myotonic dystrophy
DMD	Duchenne muscular dystrophy
DMPK	dystrophia myotonic protein kinase
DNA	deoxyribonucleic acid
dsRNA	double-stranded RNA
EAP	expanded access program
fALS	familial ALS
FDA	Food and Drug Administration
FKRP	fukutin-related protein
FLTD-TDP	frontotemporal lobar degeneration with TDP-43 inclusions
FTD	frontotemporal dementia
FUS	fused in sarcoma
GRT	gene-replacement therapy
HGF	hepatocyte growth factor
ILI	isolated limb infusion
IM	intramuscular
ISS-N1	intronic splicing silencer-N1
IT	intrathecal
IV	intravenous
LGMD	limb-girdle muscular dystrophy
mRNA	messenger RNA
miRNA	microRNA
OLE	open-label extension
PMO	phosphorodiamidate morpholino oligomer
polyQ	polyglutamine
PS	phosphorothioate
RISC	RNA-induced silencing complex
RNA	ribonucleic acid
RNAi	RNA interference
SMA	spinal muscular atrophy
SMN	survival of motor neuron
siRNA	small interfering RNA
SOD1	superoxide dismutase 1
TARDBP	TAR DNA-binding protein
TDP-43	transactive response DNA-binding protein 43
tMCK	muscle-specific creatine kinase (promoter)
UTR	untranslated region
vg	viral genome
2′-O-Me	2′-O-Methyl
2′-O-MOE	2′-O-methoxy ethyl/phosphorothioate
